# The cytoplasmic expression of FSTL3 correlates with colorectal cancer progression, metastasis status and prognosis

**DOI:** 10.1111/jcmm.17690

**Published:** 2023-02-18

**Authors:** Chien‐Hsiu Li, Chih‐Yeu Fang, Ming‐Hsien Chan, Chi‐Long Chen, Yu‐Chan Chang, Michael Hsiao

**Affiliations:** ^1^ Genomics Research Center Academia Sinica Taipei Taiwan; ^2^ National Institute of Infectious Diseases and Vaccinology National Health Research Institutes Miaoli Taiwan; ^3^ Department of Pathology, School of Medicine, College of Medicine Taipei Medical University Taipei Taiwan; ^4^ Department of Pathology Taipei Medical University Hospital Taipei Taiwan; ^5^ Department of Biomedical Imaging and Radiological Sciences National Yang Ming Chiao Tung University Taipei Taiwan; ^6^ Department of Biochemistry Kaohsiung Medical University Kaohsiung Taiwan

**Keywords:** bioinformatics, colorectal cancer, follistatin‐like, FSTL3, metastasis

## Abstract

Follistatin‐like (FSTL) family members are associated with cancer progression. However, differences between FSTL members with identical cancer types have not been systematically investigated. Among the most malignant tumours worldwide, colorectal cancer (CRC) has high metastatic potential and chemoresistance, which makes it challenging to treat. A systematic examination of the relationship between the expression of FSTL family members in CRC will provide valuable information for prognosis and therapeutic development. Based on large cohort survival analyses, we determined that FSTL3 was associated with a significantly worse prognosis in CRC at the RNA and protein levels. Immunohistochemistry staining of CRC specimens revealed that FSTL3 expression levels in the cytosol were significantly associated with a poor prognosis in terms of overall and disease‐free survival. Molecular simulation analysis showed that FSTL3 participated in multiple cell motility signalling pathways via the TGF‐β1/TWIST1 axis to control CRC metastasis. The findings provide evidence of the significance of FSTL3 in the oncogenesis and metastasis of CRC. FSTL3 may be useful as a diagnostic or prognostic biomarker, and as a potential therapeutic target.

## 1 INTRODUCTION

Malignant colorectal cancer (CRC) is a major public health problem, mainly because it is the third leading cause of death for humans.^1^ The incidence of CRC has increased in many Asian countries over the last decade.^2^ However, despite major advances in cancer screening and treatment, survival rates remain low, especially in patients with advanced stages of the disease. After initial treatment, cancer‐related morbidity and mortality are primarily caused by tumour recurrence and distant metastasis. Treatment of CRC includes surgery, if resectable, as well as chemotherapy and radiation, using either neoadjuvants or adjuvants to reduce tumour volume.^3^ When a patient has CRC that cannot be surgically removed, immunotherapy and chemotherapy are the primary strategies for shrinkage and stabilization of the tumour and suppression of further spread.^4^ In the past, targeted therapies, such as those targeting vascular endothelial growth factor (VEGF), VEGF receptor, proto‐oncogene B‐Raf (BRAF), epidermal growth factor receptor and immune checkpoint inhibitors, have been developed to treat metastatic CRC. The effects of these approaches on survival have been limited.^5^ According to 2019 cancer statistics, CRC patients have a 5‐year survival rate of 64%, but when the disease has metastasized, the survival rate drops to 12%.^6^ CRC will greatly benefit from unravelling the mechanism of tumour progression and discovering novel treatments and prognosticating targets.

Follistatin (FST)‐like 3 (FSTL3) glycoprotein is a member of the follistatin‐module‐containing protein family. Despite its typical classification as a secreted protein, FSTL3 is transported into the nuclei of cells.^7^ The secreted form of FSTL3 (isoform 1) exhibits a high degree of similarity to FST and is involved in the regulation of various biological effects by binding to and antagonizing members of the transforming growth factor‐beta (TGF‐β) superfamily, such as activin A, myostatin and bone morphogenetic protein 2 (BMP2).^8^, ^9^, ^10^ Secreted FSTL3 plays a role in bone formation, haematopoiesis and leukemogenesis.^11^, ^12^, ^13^ The function of the nuclear form of FSTL3 (isoform 2) is currently unknown but may be related to transcriptional regulation through interactions with MLLT10.^14^, ^15^

Aside from the biological regulation of FSTL3, there has been little information on the association between FSTL3 expression and malignancy, with only a few published studies. The expression of FSTL3 is higher in infiltrating ductal carcinomas of the breast than in normal tissue.^16^, ^17^ It has also been reported that the serum level of FSTL3 is higher in breast cancer patients.^18^ However, in a subsequent study, FSTL3 levels in breast cancer were inversely related to tumour size and nuclear grade and did not correlate with disease survival.^19^ The expression of FSTL3 in liver cancer is downregulated compared to normal liver tissue.^20^ Increased FSTL3 expression in non‐small‐cell lung cancer tissues has been described, with the expression of FSTL3 associated with poor prognosis.^21^ Chromosomal translocation of CCND1 to FSTL3 has been reported in a B‐cell lymphoma case.^22^ It remains unclear how alterations in FSTL3 may lead to tumour progression, and its role in the tumorigenesis of CRC is not well understood.

Here, we investigated FSTL3's role in the carcinogenesis of CRC. Using both RNA and protein as biomarkers, we showed that FSTL3 has significant prognostic value in CRC. Immunohistochemistry (IHC) staining of clinical specimens revealed a significant correlation between patients with high cytosolic FSTL3 expression and poor prognosis. Using a simulated molecular interaction model and clinical correlation analysis, we identified the Twist Family BHLH Transcription Factor 1 (TWIST1)/TGF‐β axis as a molecular mechanism regulated by FSTL3. The findings may guide the diagnosis and therapeutic development of patients with CRC.

## 2 MATERIALS AND METHODS

### 2.1 Evaluation of the prognostic value of FSTL3 by public databases

To determine the correlation between FSTL3 expression and patient outcomes, RNA expression microarray data for 177 patients with CRC were collected from the GSE database (GSE17536) (Table S5). These probes were used to measure the RNA expression levels of FST and FSTL proteins. The hazard ratio of FSTL family proteins and the survival curve of GSE 17536 were investigated using the PrognoScan online analysis platform.^23^ In addition, the colon adenocarcinoma data set (COAD) of The Cancer Genome Atlas (TCGA) was used for survival and clinicopathological characteristic analyses (Table S3). Based on prognostic indices calculated from beta coefficients multiplied by gene expression values, CRC samples were divided into two groups of approximately equal size (Table S2). Using Table S1, heatmaps were generated to illustrate RNA‐sequencing (RNA‐seq) intensity RNA‐seq by expectation–maximization (RSEM) values for different FSTL genes based on their highest, middle and lowest values in normal or tumour patients. To analyse the correlation between clinicopathological characteristics and FSTL3 expression, a chi‐square test was employed. Kaplan–Meier analysis was used for statistical evaluation as described below.

### 2.2 Patients and tissue samples

During the study period from 1998 to 2008, 236 patients with CRC who underwent tumour resection at the Wan Fang Hospital of Taipei Medical University were enrolled. As previously described, CRC specimens were used to construct tissue microarrays (TMAs).^24^ The protocol for the collection of tissue samples and clinical records was approved by the Institutional Review Board at Taipei Medical University (approval number WFH‐IRB‐99049). Sections 2 μM in thickness were cut from the TMA block. Clinical records provided information regarding histopathology and follow‐up. Overall survival (OS) was defined as the number of days the patient remained alive between the date of diagnosis and the last follow‐up or until death. The period of time until the last follow‐up or until death related to CRC was recorded from the date of treatment. The time was used to determine disease‐specific survival (DSS), which was defined as the percentage of time between the date of treatment and the date of local recurrence or newly diagnosed metastasis. In this study, all patients were followed up for at least 5 years or until death.

### 2.3 Immunohistochemistry

Immunohistochemistry staining of samples was performed as previously described.^24^, ^25^ Following deparaffinization and rehydration of the tissue sections, the sections were blocked with 3% hydrogen peroxide. Tris‐EDTA buffer (pH 9.0) was used for heat‐induced antigen retrieval. Incubation with primary antibody was performed overnight at 4°C. A 30‐min incubation with an anti‐mouse probe (MACH 1 Universal HRP Detection System; Biocare Medical, USA) followed by another 30‐min incubation with horseradish peroxidase‐polymer antibody was performed after the wash procedure. 3,3′‐Diaminobenzidine was added to yield a colour reaction. Haematoxylin was added to the sections, which were then dehydrated and mounted. Polyclonal FSTL3 antibody (HPA045378; Sigma‐Aldrich) was used.

### 2.4 IHC score assessment

Two pathologists who were blinded to the clinical parameters reviewed and scored the TMA sections stained with FSTL3. FSTL3 staining was assessed semi‐quantitatively, based on the intensity and percentage of positively stained cells. There were four levels of staining intensity: 0, none; 1, weak; 2, moderate and 3, strong. In each case, a final staining score of 0–1 or 2–3 indicated a low or high level of expression, respectively. The interpretations of the two pathologists did not differ significantly.

### 2.5 Molecular regulation simulation signalling network analysis

An analysis of the clinically relevant molecules of FSTL3 in COAD was conducted using three TCGA databases: PanCancer Atlas (https://gdc.cancer.gov/about‐data/publications/pancanatlas), Firehose Legacy (https://gdac.broadinstitute.org/runs/stddata__2016_01_28/data/LUAD/20160128/), and Nature 2014 (https://www.cancer.gov/about‐nci/organization/ccg/research/structural‐genomics/tcga).^26^ For molecules with molecular correlation coefficients, >± 0.3 Spearman's correlation was unified through a Venn diagram and analysed by Ingenuity Pathway Analysis (IPA) software (https://digitalinsights.qiagen.com/plugins/ingenuity‐pathway‐analysis/) to simulate the possible molecular regulation network. IPA is a molecular regulation simulation signalling network formed by combining a variety of omics data and known literature or databases to study the potential regulatory relationships between molecules of interest. Molecular regulatory relationships can be identified using the prediction legend. COAD gene correlation values were derived from GEPIA2 (http://gepia2.cancer‐pku.cn/#index) and TIMER2.0 (http://timer.cistrome.org/) websites. Molecular repeatability was determined based on wordcloud‐related websites. The distribution of genes in CRC cells was compiled from the CCLE data sets.

### 2.6 Statistical analysis

A chi‐square test was used for categorical data and the Student's *t* test was used for continuous variables to analyse the relationships between clinicopathological characteristics and FSTL3 expression. Kaplan–Meier analysis was used to calculate the curves for each of the three variables: disease‐free survival (DFS), DSS and OS. Differences among expression groups were assessed using the log‐rank test. The prognostic factors of DSS and DFS were evaluated by univariate and multivariate analyses using the Cox proportional hazards model. Statistical significance was set at *p* < 0.05.

## 3 RESULTS

### 3.1 FSTL family mRNA distribution in CRC

**FIGURE 1:**
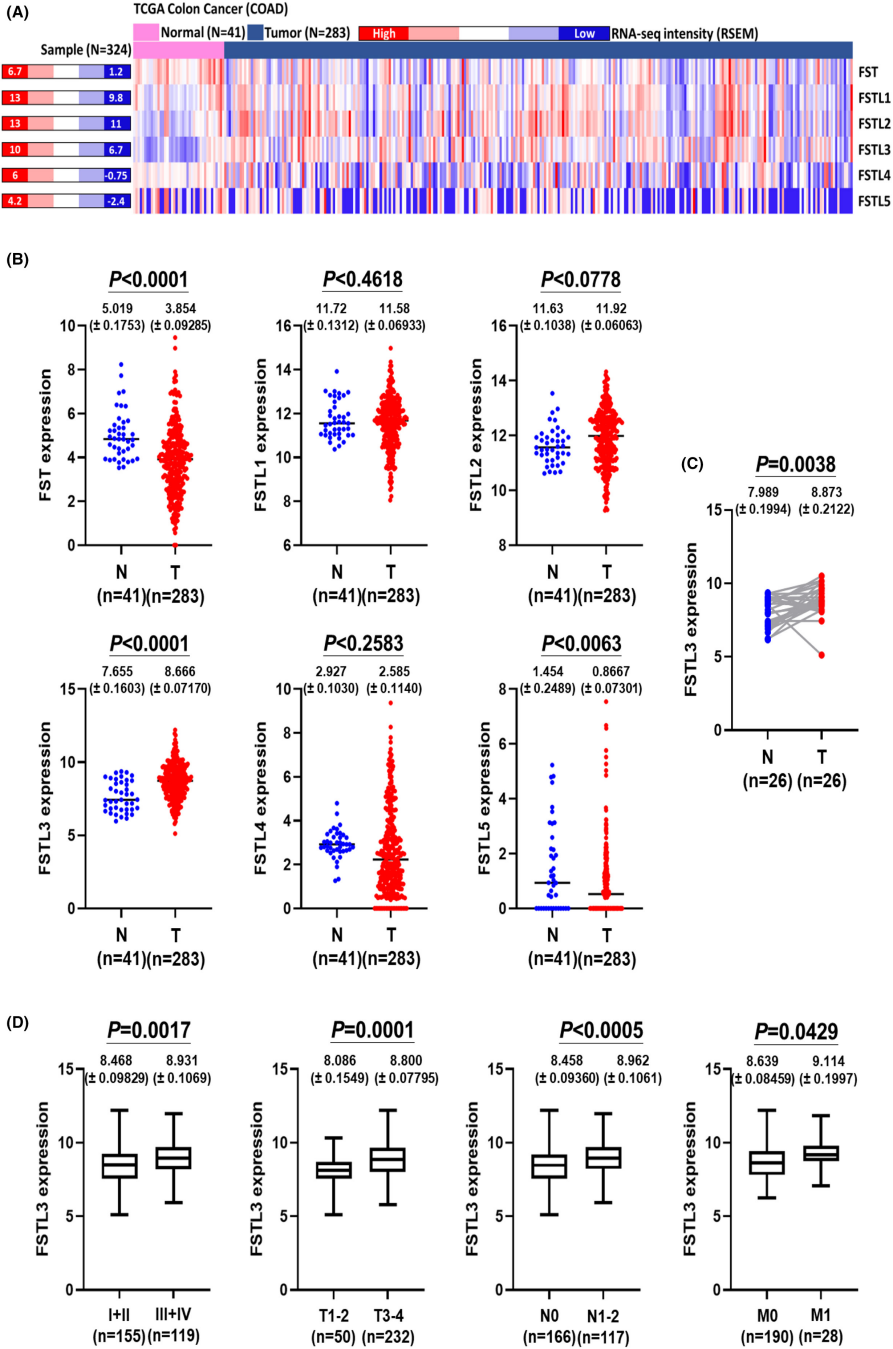
FSTL3 gene expression correlates with colorectal cancer (CRC) malignancy. (A) Heatmap illustrating the distribution of follistatin‐like (FSTL) families in CRC patients. Heatmap results represent RNA‐seq intensity RNA‐seq by expectation–maximization values for different FSTL genes based on their high to low values in different normal or tumour patients. (B) Comparison and differences between different FSTL members in patients with CRC; N: solid tissue normal (*n* = 41) and T: primary tumour (*n* = 283). (C) Paired analysis indicating differences in FSTL3 among CRC patients; N: solid tissue normal (*n* = 26) and T: primary tumour (*n* = 26). (D) Correlation between FSTL3 and different pathological stages of CRC. Tumour, node and metastasis classification was used to represent pathological stages.

The related expression levels of the FSTL components from TCGA‐COAD datasets were used to compare the expression levels of the FSTL family in CRC (Figure 1A, Table S1). Based on the comparison of the solid tissue normal and primary tumour groups, it appeared that the FSTL family and FSTL3 exhibited significant differences (*p* < 0.0001) (Figure 1B). Using different data sources (TIMER2.0), we also observed a highly significant level of FSTL3 expression in CRC (Figure S1). FSTL3 was expressed more frequently in the tumour group than in the normal group. Based on a comparison of the same cases, the expression of FSTL3 was significantly elevated in tumour tissues (*p* = 0.0038; Figure 1C, Table S2). Furthermore, FSTL3 was compared according to the differences between pathological manifestations (Table S3); it was strongly correlated with tumour stage (*p* = 0.0017), tumour size (*p* = 0.0001), degree of cancer invasion of lymph nodes (*p* < 0.0005), and metastasis (*p* = 0.0429) (Figure 1D). Overall, these comparative analyses demonstrate that FSTL3 is a unique marker of the FSTL family in CRC and that there is a positive relationship between FSTL3 and the disease process.

### 3.2 Expression level of FSTL3 mRNA is a prognostic factor for CRC

**FIGURE 2:**
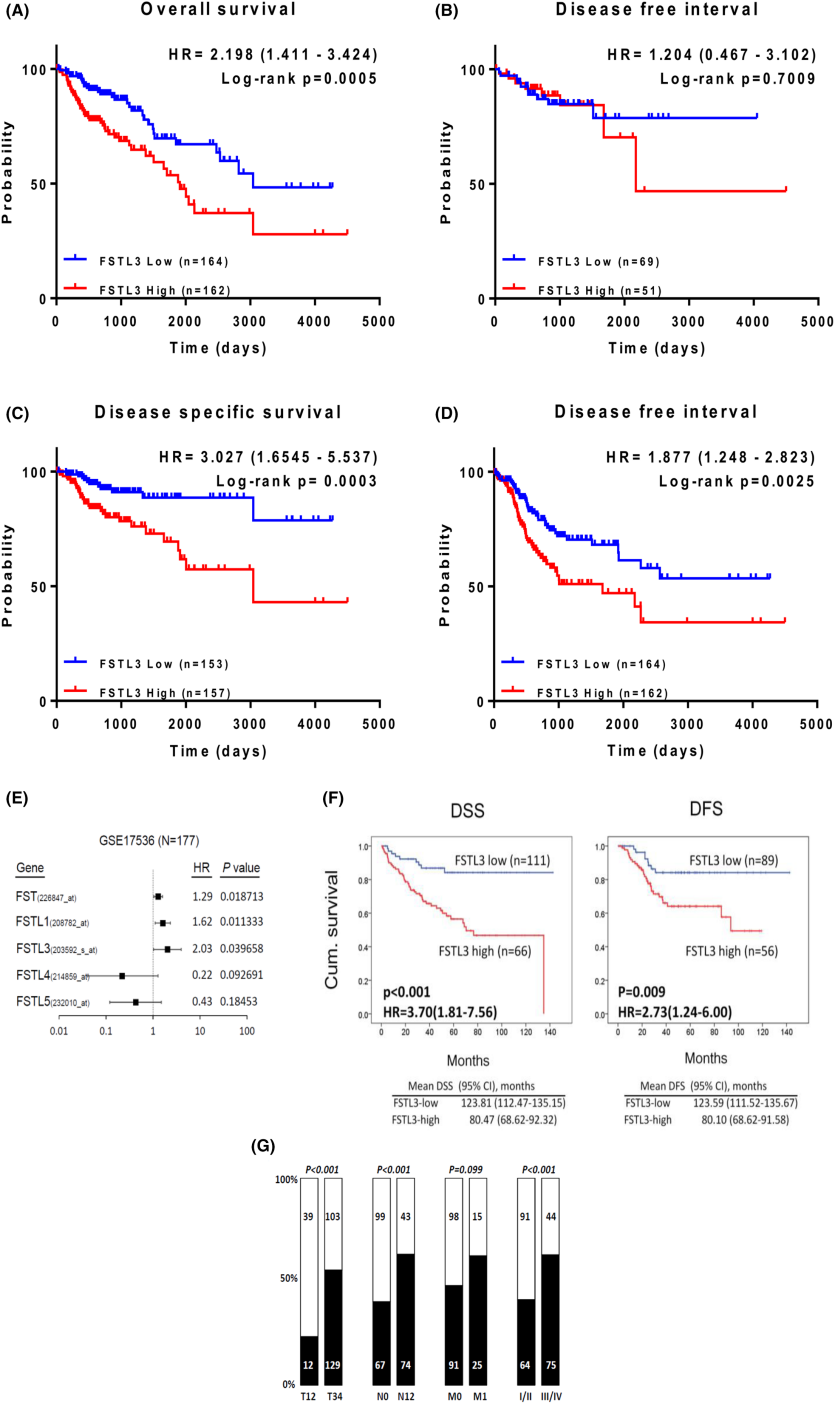
FST3 is associated with overall survival (OS), disease‐free survival (DFS) and disease‐specific survival (DSS) in colorectal cancer (CRC). (A) Association of FSTL3 with OS in CRC. (B) Correlation between FSTL3 and DFS in CRC. (C) Relationship between FSTL3 and DSS in CRC. (D) Incidence of progression‐free interval in CRC in relation to FSTL3. (E) Comparison of hazard ratios among FSTL members in GEO data set GSE 17536. (F) Analysis of DSS and DFS correlations between FSTL3 expression in GEO data set GSE 17536. (G) Relationship between FSTL3 and clinicopathological characteristics in TCGA‐COAD patients.

Using the mRNA expression data of 177 samples from CRC patients from GSE17536 (Table S5) for survival analysis, we evaluated the prognostic value of FST and FSTL family proteins in CRC. Cox proportional hazards analysis of FST and FSTL 1, 3, 4 and 5 expression revealed that FSTL3 was the most significant gene associated with survival (hazard ratio = 2.03; *p* = 0.039) in CRC patients (Figure 2E). Kaplan–Meier analysis determined that high FSTL3 expression was significantly associated with a poor DSS (*p* < 0.001; HR = 3.70 [1.81–7.56]), with a mean survival of 123.81 and 80.47 months for low and high FSTL3 groups, respectively. High FSTL3 expression was also associated with decreased DFS (*p* = 0.009; HR = 2.73 [1.24–6.00]), with a mean survival of 123.59 and 80.10 months for low and high FSTL3 groups, respectively (Figure 2F). Consistent results were observed in various data sets (SurvExpress database) (*p* = 0.003194, HR = 1.86 [1.23–2.82]) (Figure S2). This finding was further confirmed by analysing TCGA‐COAD data for the expression of FSTL3. FSTL3 expression also was associated with reduced OS (*p* = 0.0005, HR = 2.198 [1.411–3.424]) (Figure 2A), DSS (*p* = 0.0003, HR = 3.027 [1.6545–5.537]) (Figure 2C) and progression‐free interval (PFI) (*p* = 0.0025, HR = 1.877 [1.248–2.823]) (Figure 2D) in patients with CRC. However, FSTL3 expression was not associated with DFI (*p* = 0.7009, HR = 1.204 [0.467–3.102]) (Figure 2B, Table S4). Analysis of FSTL3 expression and the clinicopathological characteristics of the patients revealed a significant association between high FSTL3 expression and tumour size (T status, *p* < 0.001), lymph node invasion (N status, *p* < 0.001), and tumour stage (*p* < 0.001) (Figure 2G). Taken together, these findings suggest an increased risk of poor health outcomes associated with high expression of FSTL3 in patients with CRC who experience tumour recurrence.

### 3.3 Cytosolic FSTL3 in CRC correlates with tumour stage, distant metastasis and tumour recurrence

**FIGURE 3:**
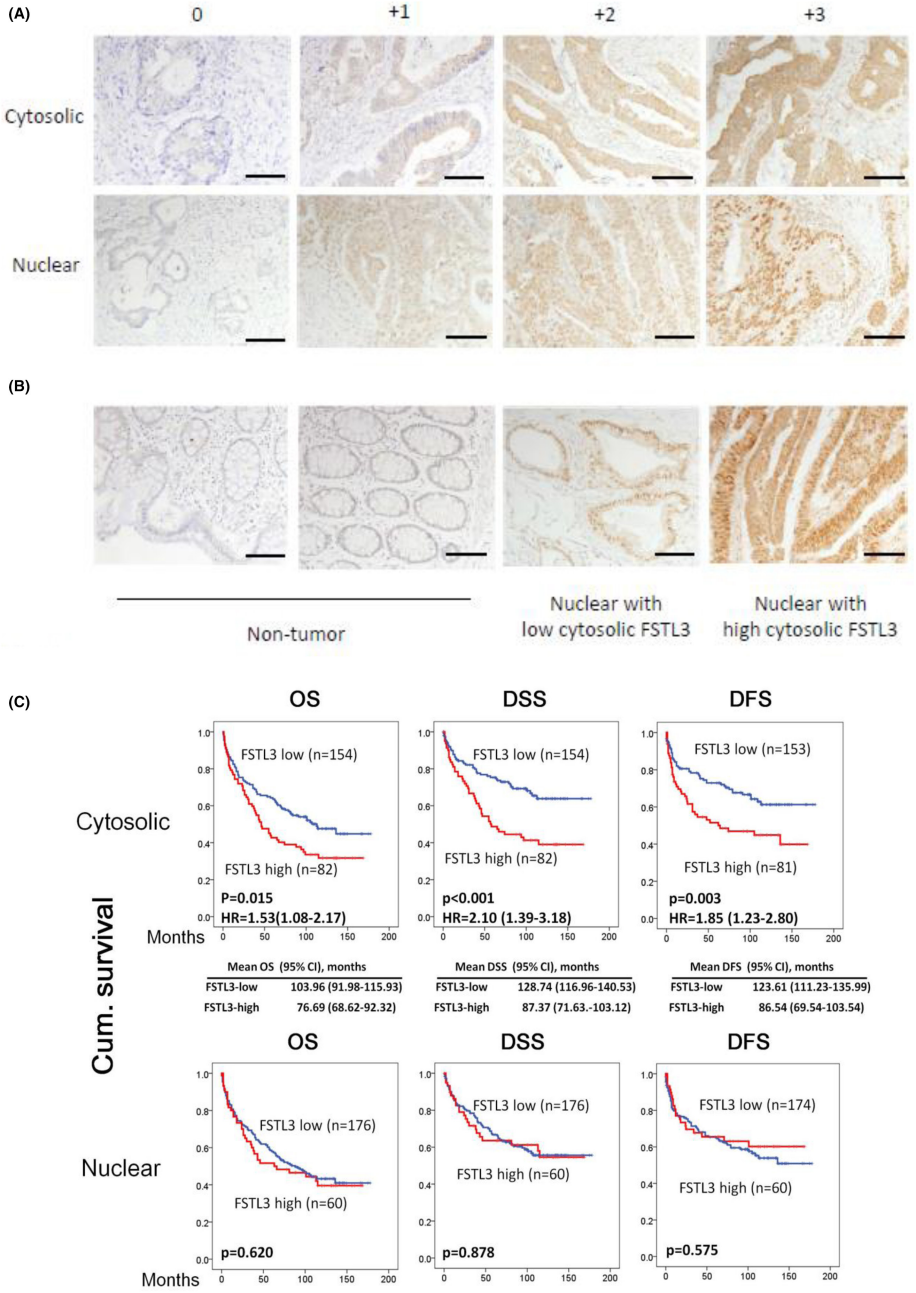
Subcellular distribution of FSTL3 protein in human colorectal cancer (CRC) tissues and association with patient outcome. Images illustrating the staining grade of FSTL3 in the cytoplasm and nuclei of cells from CRC clinical specimens. (B) Representative images of the IHC staining grade of FSTL3 in non‐tumourous and tumourous regions of CRC clinical specimens. (C) Kaplan–Meier plots illustrating the correlation of FSTL3 protein expression with overall survival (OS), disease‐free survival (DFS) and disease‐specific survival (DSS) rates in CRC patients.

We examined the mRNA expression profiles using data from public data sets. A negative association was evident between FSTL3 expression and poor prognosis among CRC patients (Figures 1 and 2). Therefore, we investigated the protein expression levels of FSTL3 in clinical CRC samples and examined whether they were correlated with patient outcomes. A total of 236 CRC patients were analysed for the expression of FSTL3. As shown in Figure 2B, FSTL3 was expressed in normal colon tissues to a greater or lesser extent (0 to +1). FSTL3 expression varied in cancer tissues (Figure 3A), ranging from no staining to strong cytoplasmic staining. In addition, nuclear staining for FSTL3 was detected in these samples (Figure 3A). The cytosolic presence of FSTL3 usually does not occur in conjunction with nuclear staining in most positively stained cases. However, weak to strong cytoplasmic FSTL3 staining was observed in samples with strong nuclear staining (Figure 3B). Based on the intensity of the positively stained cells, the expression levels of nuclear and cytosolic FSTL3 were dichotomized into low and high groups (see Methods). Eighty‐two of the 236 cancer samples stained cytologically positive for FSTL3 (34%, Table 1), while 60 of 236 cancer samples stained positively for nuclear FSTL3 (25%, Table 1). A correlation was observed between the cytosolic and nuclear staining of FSTL3 and clinicopathological features (Table 1). Sex, age, T/N status and perineural invasion did not affect the expression level of cytosolic FSTL3. In contrast, FSTL3 expression was substantially higher in patients with advanced stages (stages III and IV, *p* = 0.021), distal metastasis (*p* = 0.031) and vascular invasion (emboli, *p* = 0.048). In patients with high cytosolic FSTLR3 levels, recurrence and tumour‐specific death were significantly higher (*p* < 0.004). Conversely, nuclear FSTL3 expression was not significantly associated with any clinicopathological features associated with CRC.

**TABLE 1:** Analysis of associations between clinicopathological features and FSTL3 expression in patients with colorectal cancer.

Variables	Cytosolic FSTL3			Nuclear FSTL3		
	Low [*n* = 154]	High [*n* = 82]	*p* Value	Low [*n* = 176]	High [*n* = 60]	*p* Value
Age, years						
≤65	51 (63.7)	29 (36.3)	0.418	61 (76.3)	19 (23.7)	0.399
>65	103 (66.0)	53 (34.0)		115 (73.7)	41 (26.3)	
Gender						
Male	67 (68.3)	31 (31.7)	0.240	72 (73.5)	26 (26.5)	0.428
Female	87 (63.0)	51 (37.0)		104 (75.3)	34 (24.7)	
Stage*						
I + II	78 (72.9)	29 (27.1)	0.021	80 (82.5)	27 (17.5)	0.532
III + IV	76 (59.3)	52 (40.7)		95 (74.2)	33 (25.8)	
Tumour size (T)*						
T1 + T2	34 (68.0)	16 (32.0)	0.407	35 (70.0)	15 (30.0)	0.260
T3 + T4	120 (64.8)	65 (35.2)		140 (75.7)	45 (24.3)	
Lymph node status (N)*						
N0	82 (70.0)	35 (30.0)	0.092	86 (73.5)	31 (26.5)	0.426
N1–N3	72 (61.0)	46 (39.0)		89 (75.4)	29 (24.6)	
Distal metastasis (M)						
No	136 (68.0)	64 (32.0)	0.031	150 (75.0)	50 (25.0)	0.433
Yes	18 (50.0)	18 (50.0)		26 (72.2)	10 (27.8)	
Emboli						
No	79 (71.2)	32 (28.8)	0.048	83 (74.7)	28 (25.3)	0.534
Yes	75 (60.0)	50 (40.0)		93 (74.4)	32 (25.6)	
Perineural invasion						
No	123 (67.9)	58 (32.1)	0.079	135 (74.6)	46 (25.4)	0.562
Yes	31 (56.3)	24 (43.7)		41 (74.)	14 (25.5)	
Cancer death						
No	108 (74.5)	37 (25.5)	<0.001	108 (74.5)	37 (25.5)	0.574
Yes	46 (50.5)	45 (49.5)		68 (74.7)	23 (25.3)	
Recurrence						
No	104 (72.2)	40 (27.8)	0.004	105 (72.9)	39 (27.1)	0.283
Yes	50 (54.3)	42 (45.7)		71 (77.1)	21 (22.9)	

### 3.4 FSTL3 expression is associated with poor prognosis and high incidence of tumour recurrence in patients with CRC

Kaplan–Meier analysis and log‐rank test were used to determine the prognostic value of FSTL3 expression in CRC. Patients with high FSTL3 expression displayed reduced OS (*p* = 0.015; HR = 1.53 [1.08–2.17]), with a mean survival of 103.96 and 76.69 months for the low FSTL3 and high FSTL3 groups, respectively. High FSTL3 expression was also associated with DSS (*p* = 0.001; HR = 2.10 [1.39–3.18]), with a mean survival of 128.74 and 87.37 months for the low and high FSTL3 groups, respectively. Finally, FSTL3 expression was associated with DFS (*p* = 0.003; HR = 1.85 [1.23–2.80]), with a mean survival of 123.61 and 86.54 months for the low and high FSTL3 group, respectively. In these analyses, the nuclear expression of FSTL3 did not significantly contribute to the prognosis of CRC patients (Figure 3C). Therefore, the following analyses present only the results of the cytosolic FSTL3 staining assay. The prognostic factors of CRC patients were analysed using Cox proportional hazards regression analysis. According to univariate analyses of DSS, tumour T status, lymph node status, distal metastases, tumour stage, vascular invasion (emboli), perineural invasion, and FSTL3 expression were significant predictors of poor outcomes (*p* < 0.05; Table 2). The expression of FSTL3 was a significant prognostic marker in multivariate analysis of DSS, along with tumour T status and distal metastasis. The risk of death in DSS was 1.67‐fold higher in patients with high FSTL3 expression (95% confidence interval [CI] 1.08–2.58 and; *p* = 0.020). FSTL3 expression and tumour T status were significant predictors, as were lymph node status, distal metastasis, tumour stage, vascular invasion (emboli), and perineural invasion in the univariate analyses (*p* < 0.05; Table 2). However, only distal metastasis was significant. Together, these findings suggest that FSTL3 is a biomarker for adverse outcomes, DSS, and DFS in patients with CRC.

**TABLE 2:** Analysis of disease‐specific survival (DSS) and disease‐free survival (DFS) of colorectal cancer patients by univariate and multivariate models.

Variables	Univariate analysis				Multivariate analysis			
	DSS		DFS		DSS		DFS	
	HR (95% CI)	*p* Value	HR (95% CI)	*p* Value	HR (95% CI)	*p* Value	HR (95% CI)	*p* Value
Age, years								
≤65	1		1		1		1	
>65	1.28 (0.82–1.98)	0.280	1.12 (0.73–1.75)	0.953	1.42 (0.89–2.26)	0.141	1.19 (0.75–1.87)	0.457
Sex								
Male	1		1		1		1	
Female	0.91 (0.60–1.38)	0.638	0.92 (0.60–1.39)	0.677	0.93 (0.60–1.44)	0.745	0.79 (0.51–1.22)	0.296
AJCC stage								
I + II	1		1		1		1	
III + IV	2.78 (1.75–4.41)	<0.001	3.52 (2.18–5.70)	<0.001	1.14 (0.45–2.88)	0.776	2.18 (0.87–5.43)	0.095
Tumour T status								
T1 + T2	1		1		1		1	
T3 + T4	2.66 (1.38–5.13)	0.004	4.85 (2.12–11.10)	<0.001	1.79 (0.90–3.56)	0.100	3.42 (1.46–8.01)	0.005
Lymph node metastasis								
No	1		1		1		1	
Yes	1.38 (1.11–1.71)	0.003	1.48 (1.19–1.83)	<0.001	1.18 (0.755–1.83)	0.475	0.83 (0.55–1.25)	0.377
Distal metastasis								
No	1		1		1		1	
Yes	11.5 (7.06–18.7)	<0.001	15.0 (8.84–25.60)	<0.0.001	9.03 (4.93–16.54)	<0.0.001	9.06 (4.91–16.72)	<0.0.001
Emboli								
Negative	1		1		1		1	
Positive	2.00 (1.29–3.10)	0.002	2.65 (1.68–4.18)	<0.001	1.13 (0.66–1.91)	0.660	1.41 (0.83–2.42)	0.203
Perineural invasion								
No	1		1		1		1	
Yes	1.80 (1.16–2.78)	0.009	1.98 (1.28–3.07)	0.002	1.08 (0.63–1.82)	0.786	1.22 (0.78–2.02)	0.436
FSTL3								
Low	1		1		1		1	
High	2.10 (1.39–3.18)	<0.001	1.85 (1.23–2.80)	0.003	1.67 (1.08–2.58)	0.020	1.42 (0.91–2.17)	0.128

Abbreviations: AJCC, American Joint Committee on Cancer, eight editions; CI, confidence interval; HR, Hazard ratio.

### 3.5 TGF‐β signalling is involved in FSTL3‐mediated CRC progression

**FIGURE 4:**
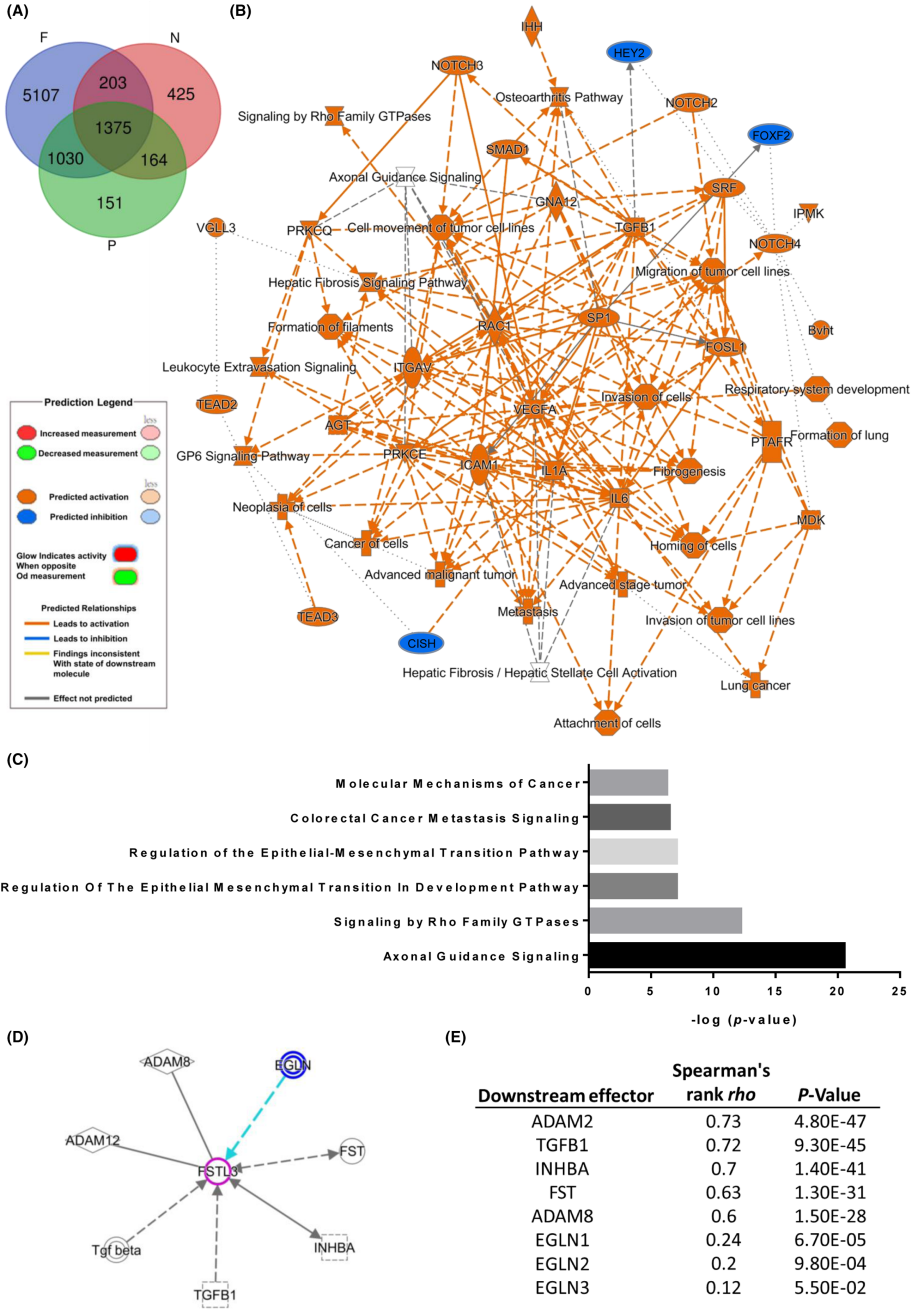
Simulation of the role of FSTL3 in the progression of colorectal cancer (CRC). (A) Venn diagram of molecules related to FSTL3 across multiple data sets. (B) Gene ontology findings and possible molecular networks associated with highly related molecules of FSTL3. (C) Signalling pathways involved in FSTL3‐related molecules. (D) Interactions between FSTL3 and downstream effectors in CRC. (E) Correlation between downstream effectors and FSTL3.

Molecular regulation simulation signalling network analysis was performed as previously described^26^ to determine the molecular mechanism through which FSTL3 mediates CRC progression. TCGA CRC data sets were downloaded to identify common molecules related to FSTL3 using a Venn diagram (Figure 4A, Table S6). The Venn diagram revealed a Spearman's correlation of more than ±0.3 related to FSTL3 (Figure 4B). IPA assessment was performed on these consistent molecules to generate simulated molecular networks associated with possible biological functions (Figure 4B), and possible underlying signalling pathways (Figure 4C, Table S7). Gene ontology previously implicated FSTL3 in a wide variety of signalling pathways associated with epithelial–mesenchymal transition (EMT), including the signalling pathway involved in cancer metastasis. The finding is consistent with our analysis of FSTL3 and distal metastases based on pathological features (Table 2). Unbiased analysis of molecular regulation identified molecules that have been reported to be involved in the regulation of FSTL3, including TGF‐β1, INHBA, ADAM12, ADAM8, FST, EGLN1, EGLN2 and EGLN3, which were linked to a simulated model (Figure 4D). Several molecules were positively related to FSTL3, including TGF‐β1, INHBA, ADAM2, ADAM8 and FST (Figure 4E). Among these, TGF‐β1, FST and INHBA function as upstream regulators of FSTL3. TGF‐β1 was the most relevant molecule (*R* = 0.72). Figure S3 displays the molecular map of TGF‐β1 signalling that may be activated by FSTL3 (Figure 3A) and the molecules regulated by TGF‐β1 (Figure S3B). Analyses of CCLE revealed a correlation between FSTL3 and TGF‐β1 expression in CRC cells (*R* = 0.443) (Figure S4, Table S8). The collective findings demonstrate that FSTL3 can contribute to CRC metastasis through EMT‐related signalling and possibly through the TGF‐β1 pathway.

### 3.6 TWIST1 as a transcription regulator connects the TGF‐β/FSTL3 axis in CRC

**FIGURE 5:**
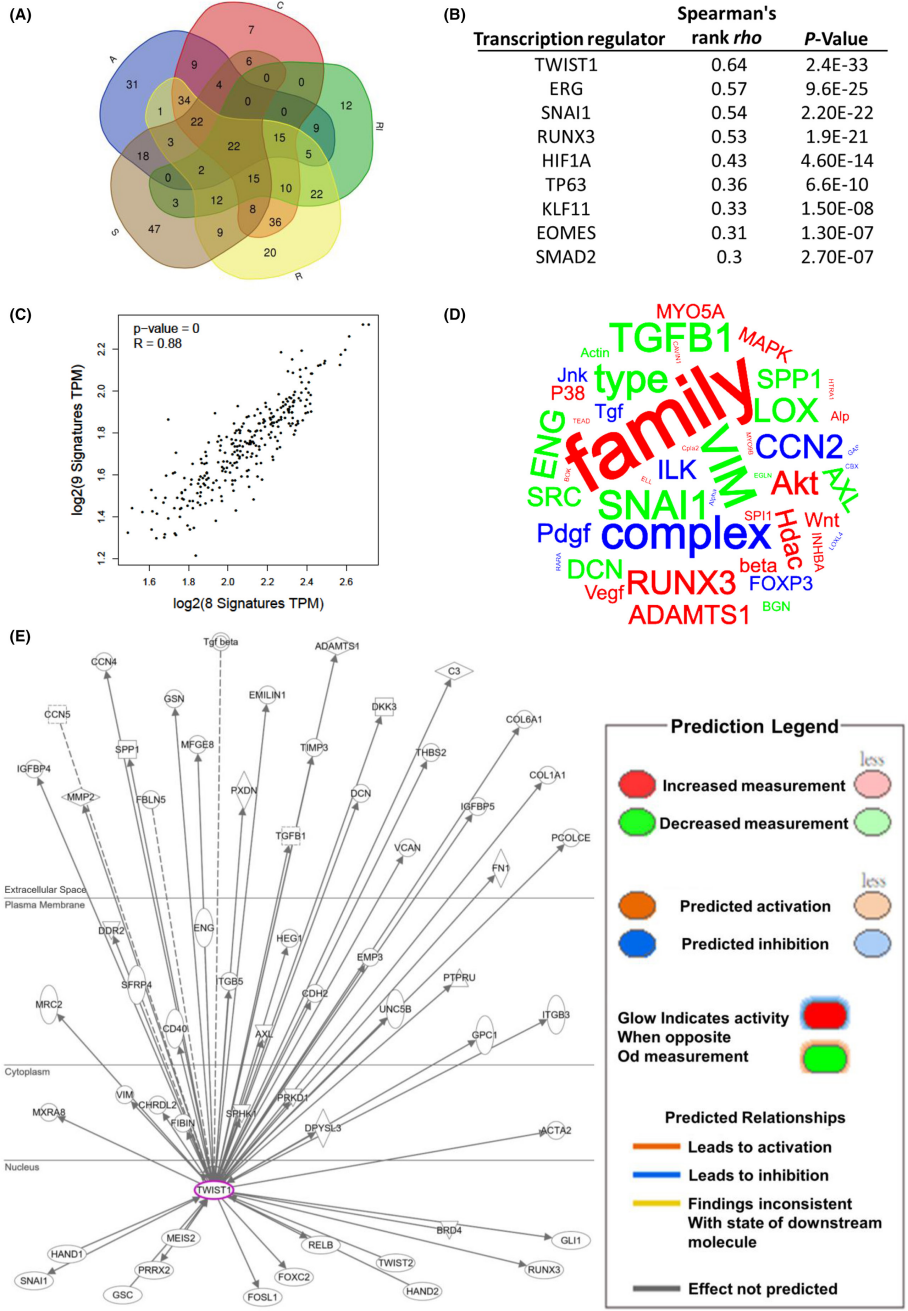
TWIST1 is involved in cell motility mediated by FSTL3. (A) Venn diagram of molecules participating in similar signals associated with different epithelial–mesenchymal transition (EMT) motility behaviours. (B) Upstream regulators that are highly relevant to FSTL3 in colorectal cancer (CRC). (C) Regulator downstream or upstream of FSTL3 has a highly positive signature in CRC. (D) Wordclouds analysis reveals molecules that are primarily related to EMT signalling pathways. (E) Molecular network of TWIST1 illustrates the involvement of FSTL3‐related molecules in CRC.

**FIGURE 6:**
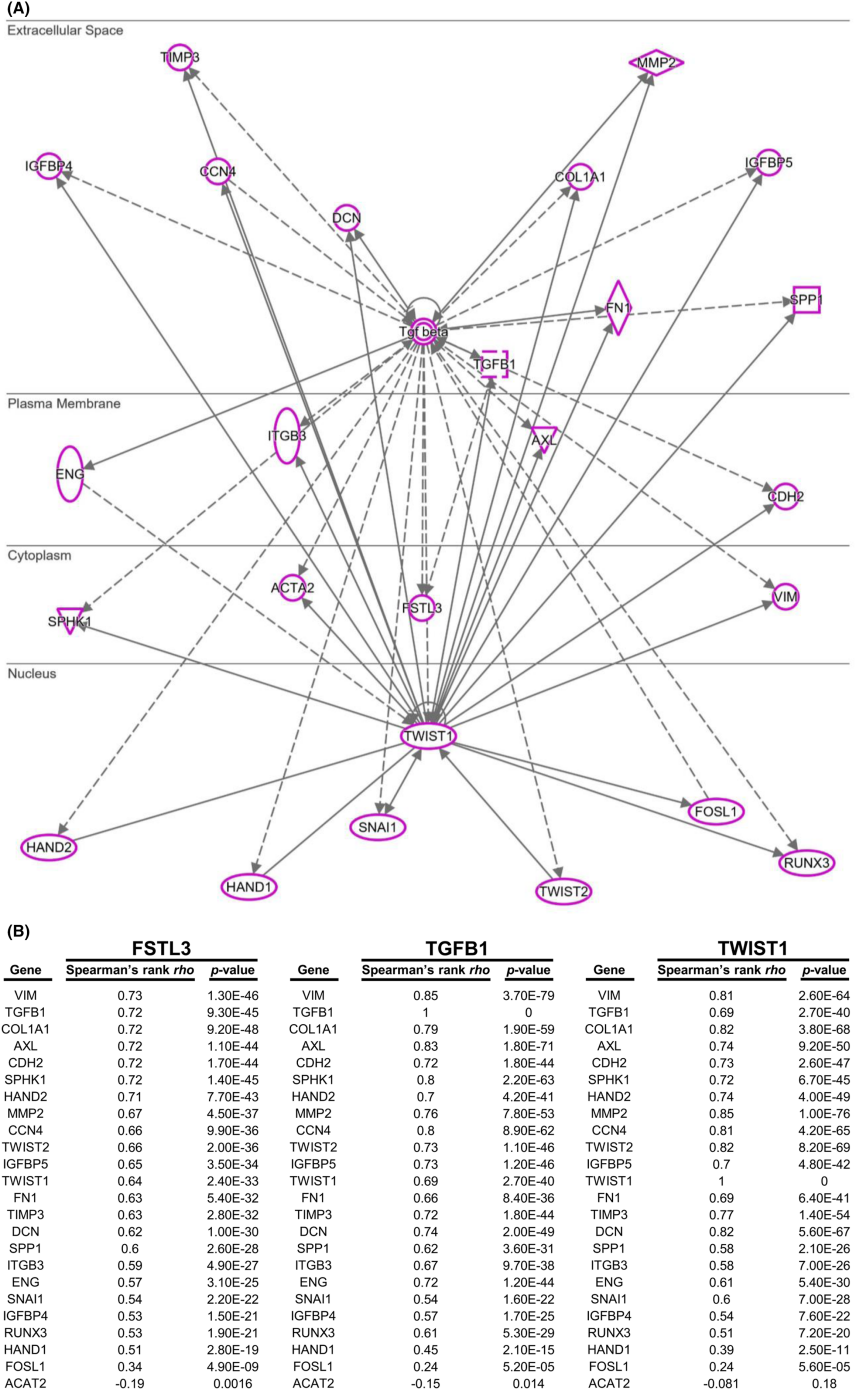
FSTL3‐TGFβ‐TWIST1 axis is positively correlated in colorectal cancer (CRC). (A) Molecule regulation map illustrating the relationship between FSTL3, TGFβ and TWIST1. (B) Strong Spearman's correlation between molecules controlled by the TGF‐β‐TWIST1 axis and molecules controlled by FSTL3 in CRC in the GEPIA2 database.

To further investigate the important transcriptional regulators that influence EMT‐related signalling through FSTL3, a Venn diagram analysis was performed on the molecules affected by these EMT‐related signalling mechanisms (Figure 5A, Table S9). IPA identified many transcription regulators related to FSTL3 regulation, including TWIST1, ERG, SNAI1, RUNX3, HIF1A, TP63, KLF11, EOMES, and SMAD2 (Figure 5B). A strong relationship was evident between these nine transcription factors and the eight molecules that are regulated by FSTL3 (*R* = 0.88) (Figure 5C). The finding supported the unbiased analysis results (Figure 4D,E). Statistically, these EMT‐related molecules, including SNAI1, VIM and TGF‐β1, were mostly affected by FSTL3 (Figure 5D, Table S10). These factors were correlated with the TWIST1 and FSTL3 transcription regulators, which were the most correlated molecules (*R* = 0.64) (Figure 5B). Consistently, CCLE analysis revealed that FSTL3 was associated with TWIST1 in different CRC cells (*R* = 0.601) (Figure S5A, Table S11). TWIST1 regulated the molecular network of FSTL3‐related molecules (Figure 5E). A combined analysis of the IPA results (Figures 4D and 5E, Figure S3) was performed to determine the molecules regulated by TGF‐β, FSTL3 and TWIST1 (Figure 6A). The correlation of these molecules with FSTL3, TGF‐B1 and TWIST1 was evident (Figure 6B). This is noteworthy given that each of these molecules had a Spearman's correlation >0.3, with the exception of FOSL1 and ACAT2. The majority of these correlations in patients were observed in cell lines associated with CRC (Figure S6). Notably, after considering the correlation of OS with the TGF‐β/FSTL3/TWIST1 axis and TWIST1 downstream effectors, patients with TGF‐β (*p* = 0.00093) or TWIST1 (*p* = 0.0012) had an increased risk of developing FSTL3 (*p* = 0.006) (Figure S7). In patients with TWIST1, downstream regulators (Table S12), such as RUNX3 (*p* = 0.0012), IGFBP4 (*p* = 0.0015), HAND2 (*p* = 0.0022), CCN4 (*p* = 0.0026), VIM (*p* = 0.0027), SNAI1 (*p* = 0.0029), TIMP3 (*p* = 0.0053) and MMP2 (*p* = 0.0057), increased the risk of FSTL3 (*p* = 0.006) (Figure S7). The collective findings indicate that the TGF‐β1‐FSTL3‐TWIST1 axis could play a significant role in the EMT of CRC cells.

## 4 DISCUSSION

Metastasis is the primary cause of bottlenecks in the treatment of cancers, including CRC. Several studies have demonstrated a direct relationship between cancer cell metastasis and poor prognosis in CRC patients. In light of the heterogeneity of cancer, it is critical to identify specific markers for diagnosis and therapeutic targets. Big data can also be utilized to identify suitable markers for diagnosis. Accordingly, in the present study, the distribution of FSTL family members in CRC was studied using published data and compared according to their clinical relevance. In this study, we discovered that FSTL3 may serve as a biomarker in the FSTL family, which is unique to CRC. IHC analysis supported the results of in silico analysis of FSTL3, with a correlation between increased expression of FSTL3 and adverse outcomes in CRC patients, including OS, DFS, PFS and DSS.

The data we analysed are consistent with previous research on related topics.^27^, ^28^, ^29^ Our previous analyses of FSTL1 in lung cancer have shown that its location within the cell may have a significant effect on its function.^30^, ^31^ Thus, in this study, we sought to determine whether the distribution of FSTL3 in tissues is associated with different clinical significance. IHC demonstrated that only cytosolic FSTL3 was correlated with CRC‐related prognosis outcome, rather than nuclear FSTL3. Notably, the pathological features indicated a strong correlation between cytosolic FSTL3 levels and distal metastases (Table 2). The present results will inform the interpretation of FSTL3 expression in future clinical prognostication efforts.

A key factor in metastasis is the ability of a cell to undergo EMT. Thus, EMT is considered a hallmark of cancer progression. EMT‐related molecules are used as markers for defining the relative morphology of cells and as targets for the inhibition of cancer metastasis. Using simulated analysis, we discovered that FSTL3‐related molecules are involved in multiple signalling pathways that regulate cell migration. These results support the clinical finding that cytosolic FSTL3 is associated with distant metastases. These pathways include axonal guidance signalling, signalling by Rho family GTPases, EMT and the role of EMT in development, and CRC metastasis signalling. These important signalling pathways have been implicated in CRC metastasis.

In addition, analysis of upstream regulators also identified many transcription factors involved in EMT, including TWIST1 and SNAI1, which have been implicated in CRC malignancy.^32^, ^33^ Based on this correlation, we propose that TWIST1 is the main molecule affected by FSTL3 expression. This suggestion is supported by the performance of TWIST1 dominant SNAI1 and VIM in the molecular regulation network. The correlation between FSTL3 and TWIST1 was further evidenced in CRC‐related cell lines. The expression of FSTL1, TWIST1 and relevant downstream molecules, such as SNAI1 and VIM, was highly correlated in CRC cells. The expression of VIM in CRC tissues has reportedly been correlated with TGF‐β/SMAD2, particularly phosphorylation‐SMAD2.^34^ Our analysis indicated that FSTL3 was highly correlated with VIM and positively correlated with SMAD2. Therefore, the present study data support previous findings and strongly suggest that FSTL3 regulates EMT, possibly in conjunction with TWIST1, as a mechanism for CRC metastasis.

FSTL3 is involved in macrophage infiltration.^27^ In particular, FSTL3 levels have been correlated with M2 macrophages. FSTL3 overexpression results in an increase in macrophage‐associated markers, such as CD206 and CD163. Furthermore, TGF‐β1 stimulates the EMT of tumour cells by M2 macrophages.^35^, ^36^ In the present study, FSTL3 was also highly correlated with TGF‐β1 in CRC (*R* = 0.71). Such a correlation was also evident in CRC‐associated cell lines. IPA revealed that many FSTL3‐related molecules participate in the regulation of TGF‐β1 signalling. In this study, FSTL3 promoted the reprogramming of EMT and the microenvironment through TGF‐β1. As expected, the upstream regulators also identified SMAD2 downstream of TGF‐β1 and associated SMAD2 with FSTL3 expression. In particular, we identified TWIST1 (*R* = 0.64) as the transcription factor most associated with FSTL3. The signalling map of TWIST1 indicated that it dominated TGF‐β1. Taking this correlation into consideration, it was found that FSTL1, TGF‐β1 and TWIST1 were all highly correlated in CRC. We suggest that FSTL3 regulates TGF‐β1 via TWIST1. It is noteworthy that TWIST1 and SNAI1 expression in CRC tissue has previously been correlated with TGF‐β1 and is reflected in the prognostic data.^32^ Therefore, our analysis is sufficient to reflect past observations and to provide evidence of a possible regulatory relationship between FSTL3 and TWIST1/TGF‐β1.

TGF‐β has previously been shown to regulate FSTL1 expression. As we previously discovered, FSTL1 can regulate the progression of lung cancer by interacting with secreted phosphoprotein 1 (SPP1), and the distribution of molecular molecules associated with BMP and TGF‐β signalling is the opposite. In gut tissues, fibrosis and carcinogenesis are highly correlated with the stability of TGF‐β signalling.^37^ Based on this study, FSTL3 appeared to be positively correlated with TGF‐β in both tissue and cellular data. Consequently, different FSTL family members have disparate roles and are regulated differently in different tissues. It remains to be determined whether FSTL3 also participates in BMP signalling in CRC and whether it cross‐talks with TGF‐β signalling.

In conclusion, the findings of the present study confirm the clinical significance of cytosolic FSTL3 in CRC progression and explain the molecular mechanism of FSTL3. The findings also highlight the potential of FSTL3 as a specific target through big data screening and validation of clinical organization.

## AUTHOR CONTRIBUTIONS

**Chien‐Hsiu Li:** Conceptualization (equal); data curation (equal); formal analysis (equal); investigation (equal); methodology (equal); project administration (equal); resources (equal); software (equal); validation (equal); visualization (equal); writing – original draft (equal); writing – review and editing (equal). **Chih‐Yeu Fang:** Conceptualization (equal); data curation (equal); formal analysis (equal); investigation (equal); methodology (equal); project administration (equal); resources (equal); software (equal); validation (equal); visualization (equal); writing – original draft (equal); writing – review and editing (equal). **Ming‐Hsien Chan:** Methodology (equal); project administration (equal); resources (equal); software (equal). **Chi‐Long Chen:** Data curation (equal); formal analysis (equal); resources (equal); software (equal); validation (equal). **Yu‐Chan Chang:** Funding acquisition (equal); supervision (equal). **Michael Hsiao:** Conceptualization (equal); funding acquisition (equal); supervision (equal); validation (equal).

## FUNDING INFORMATION

This study was supported by the Ministry of Science and Technology (MOST‐110‐2320‐B‐010‐008‐MY2), Yen Tjing Ling Medical Foundation (CI‐111‐9) and Veterans General Hospitals and University System of Taiwan Joint Research Program (VGHUST111‐G3‐3‐2) to Y‐CC.

## CONFLICT OF INTEREST STATEMENT

There are no competing interests declared by the authors.

## Supporting information


Figure S1.

Figure S2.

Figure S3.

Figure S4.

Figure S5.

Figure S6.

Figure S7.

Table S1.

Table S2.

Table S3.

Table S4.

Table S5.

Table S6.

Table S7.

Table S8.

Table S9.

Table S10.

Table S11.

Table S12.

## Data Availability

RNA‐seq data can be obtained from Gene Expression Omnibus submission SE17536.
