# Supplementary figures and images for: The cytoplasmic expression of FSTL3 correlates with colorectal cancer progression, metastasis status and prognosis
Source: J Cell Mol Med. 2023 Feb 18;27(5):672–86. doi: 10.1111/jcmm.17690 (PMC9983317; doi:10.1111/jcmm.17690)

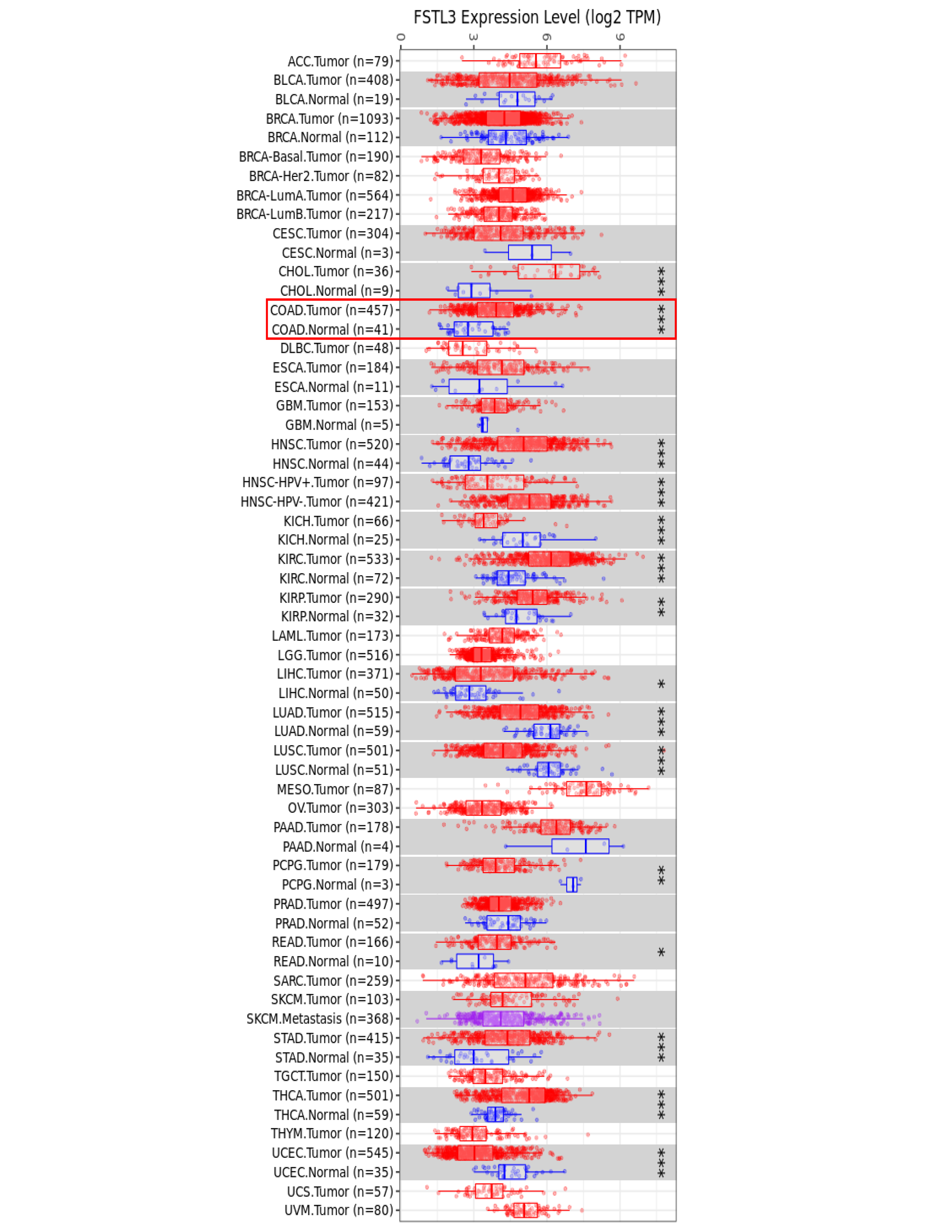

Supplement: Supplementary file 1 — Figure S1. [file JCMM-27-672-s007.tif]

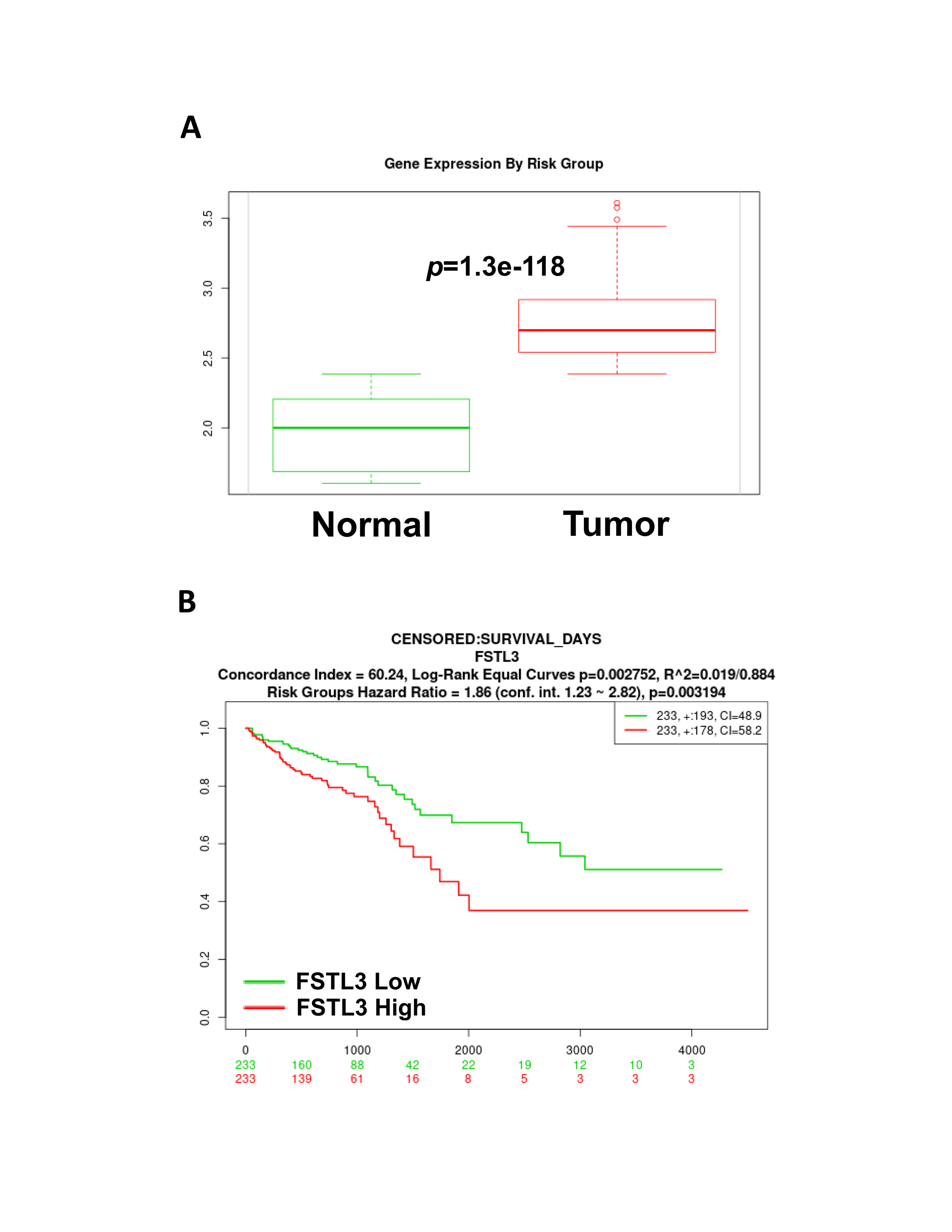

Supplement: Supplementary file 2 — Figure S2. [file JCMM-27-672-s003.tif]

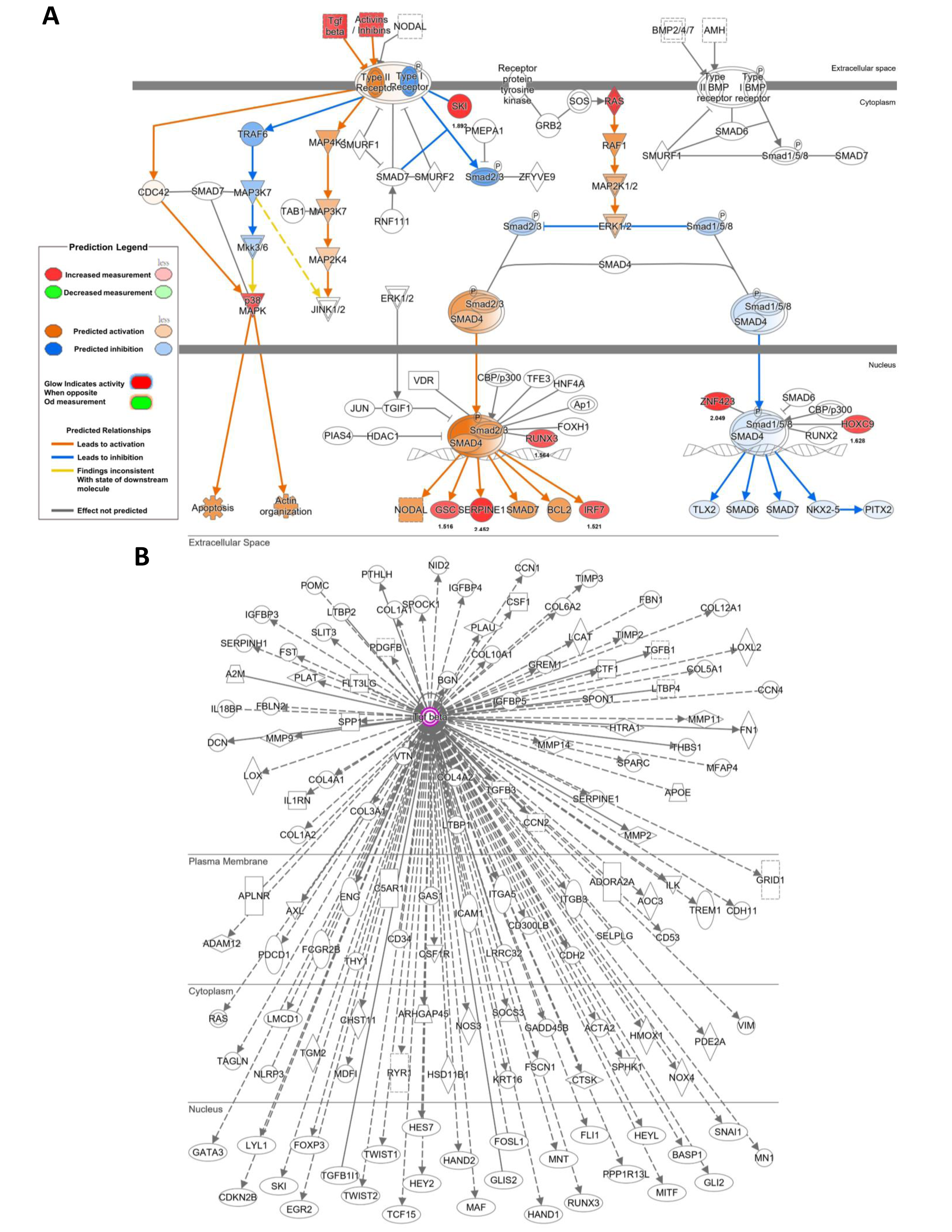

Supplement: Supplementary file 3 — Figure S3. [file JCMM-27-672-s011.tif]

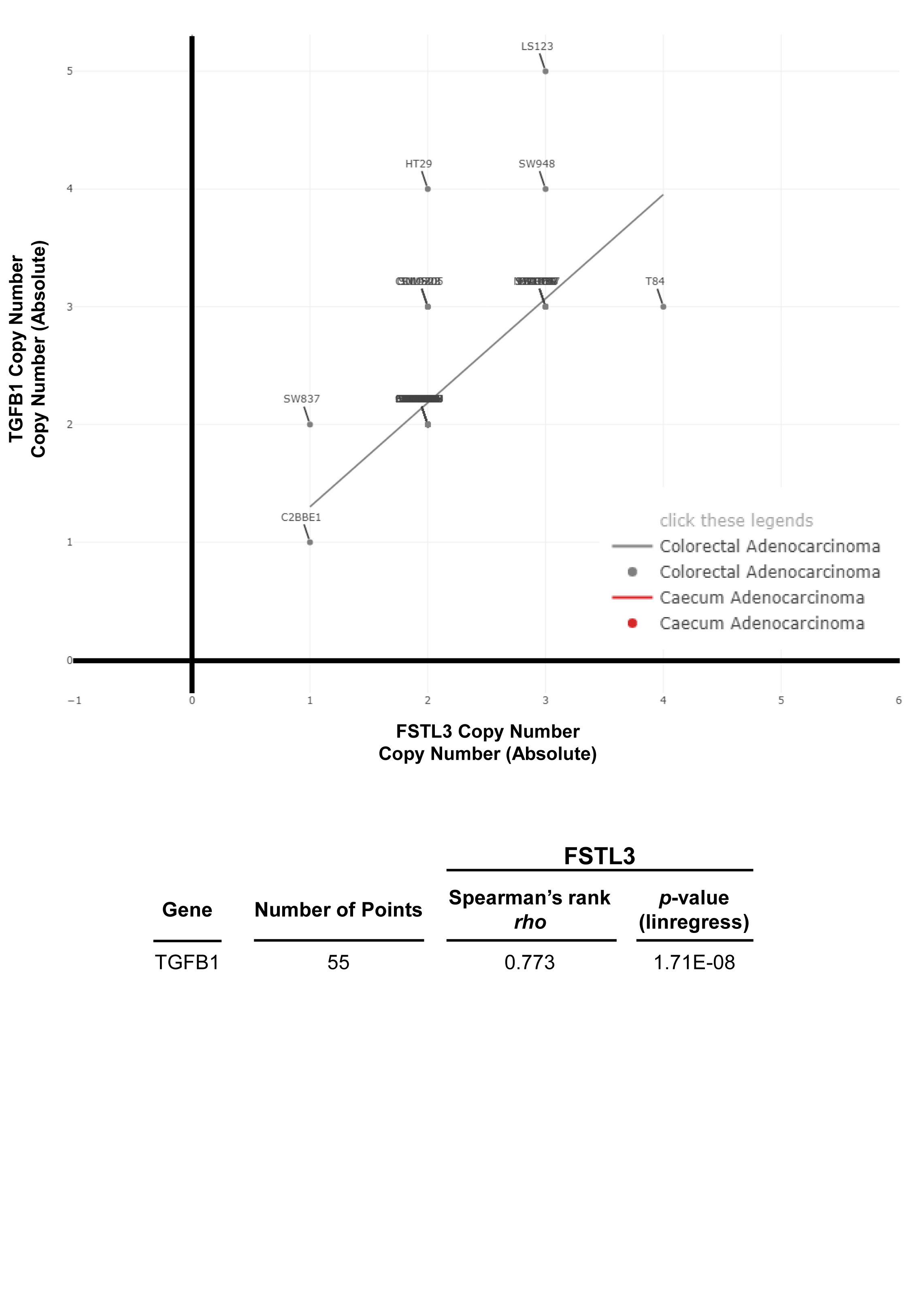

Supplement: Supplementary file 4 — Figure S4. [file JCMM-27-672-s018.tif]

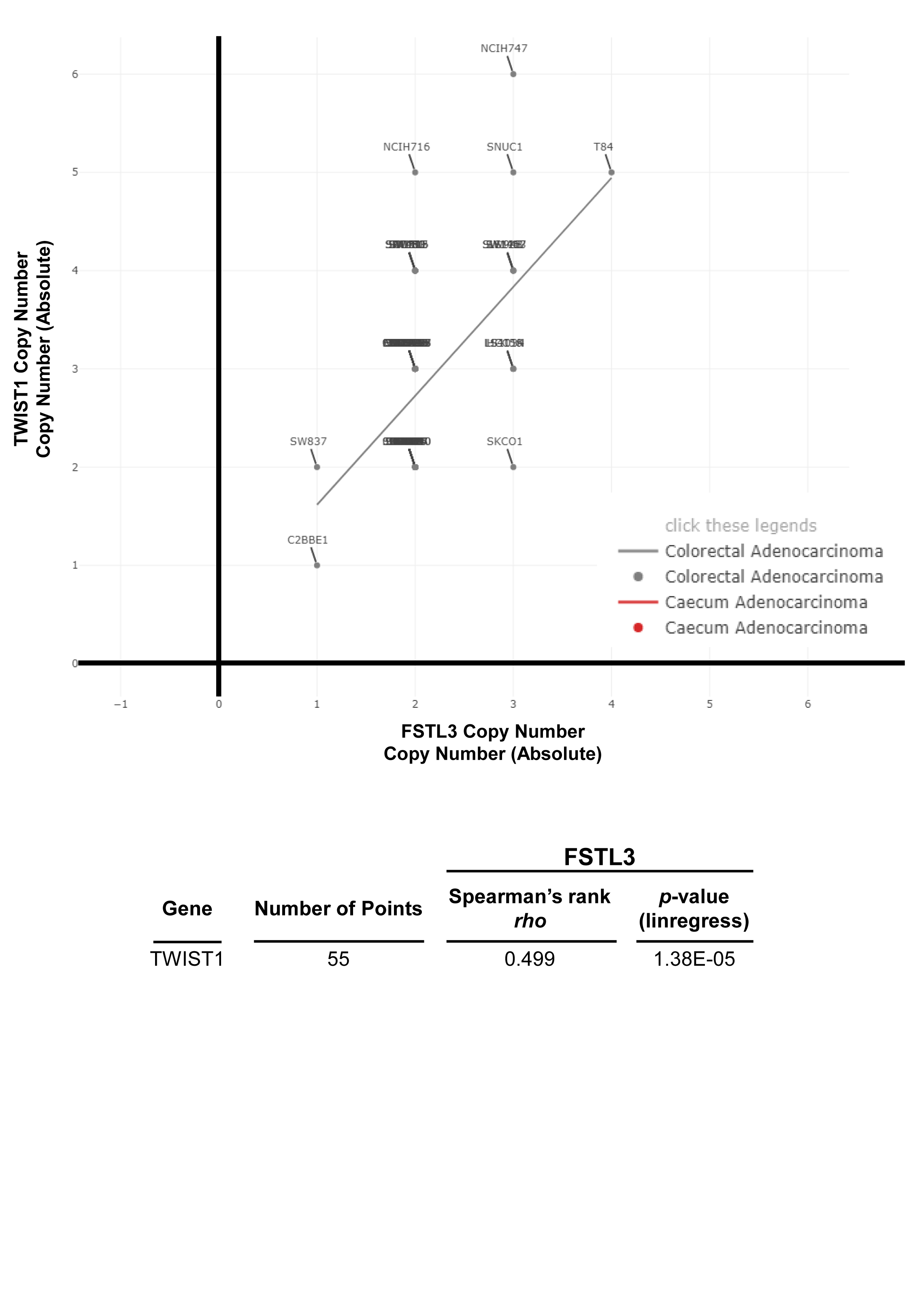

Supplement: Supplementary file 5 — Figure S5. [file JCMM-27-672-s005.tif]

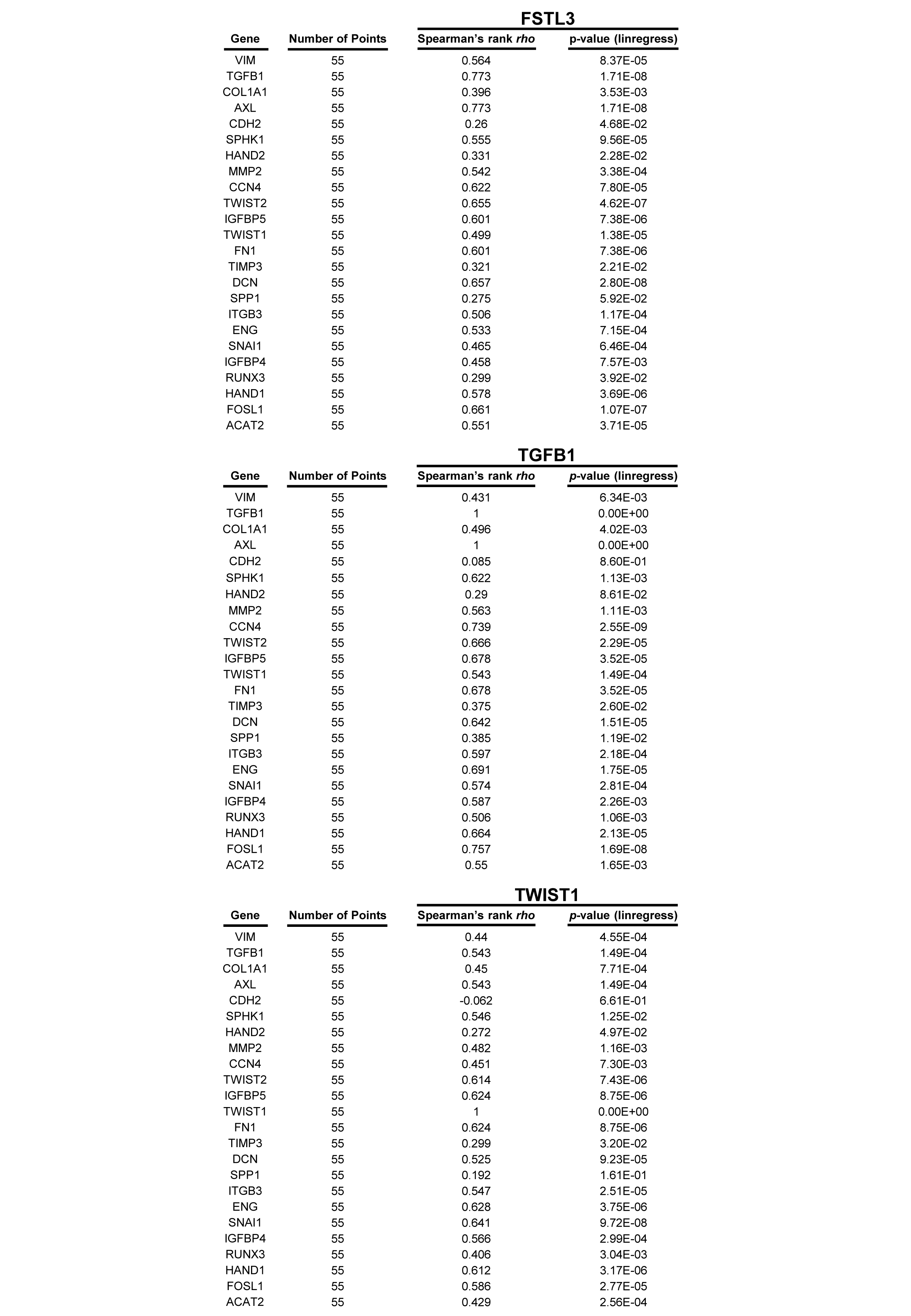

Supplement: Supplementary file 6 — Figure S6. [file JCMM-27-672-s016.tif]

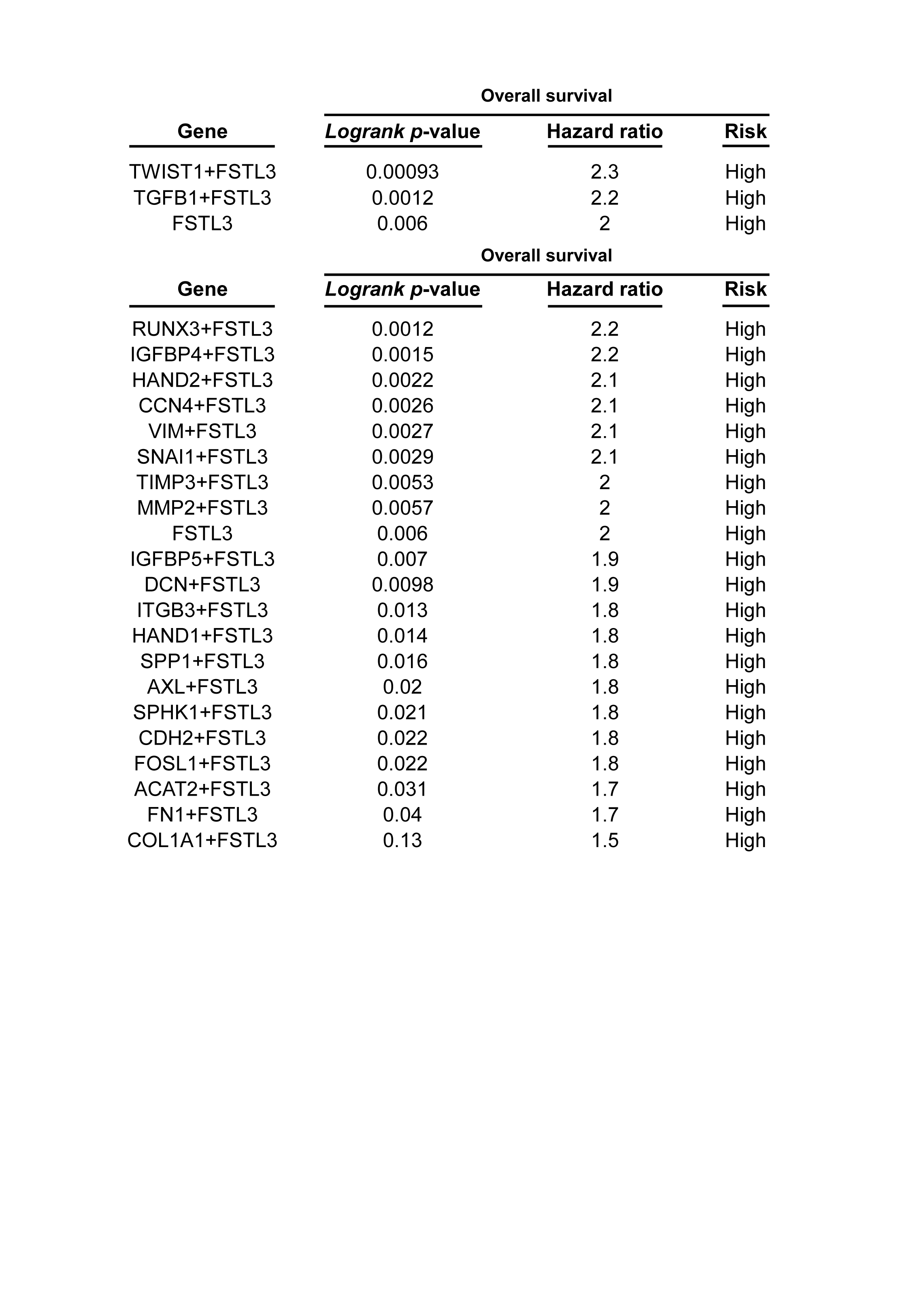

Supplement: Supplementary file 7 — Figure S7. [file JCMM-27-672-s012.tif]
